# Editorial: Natural products as potential therapies for non-alcoholic fatty liver disease

**DOI:** 10.3389/fphar.2023.1180263

**Published:** 2023-04-11

**Authors:** Islam A. A. E. H. Ibrahim, Yhiya Amen, Islam Mostafa, María De La Luz Cádiz-Gurrea

**Affiliations:** ^1^ Department of Pharmacology and Toxicology, Faculty of Pharmacy, Zagazig University, Zagazig, Al Sharqia, Egypt; ^2^ Department of Pharmacognosy, Faculty of Pharmacy, Mansoura University, Mansoura, Egypt; ^3^ Department of Pharmacognosy, Faculty of Pharmacy, Zagazig University, Zagazig, Egypt; ^4^ Department of Analytical Chemistry, Faculty of Sciences, University of Granada, Granada, Spain

**Keywords:** NAFLD, diabetes, obesity, insulin resistance, natural products

Non-alcoholic fatty liver disease (NAFLD) is the most common liver disorder worldwide with approximately 25% of the world’s population being affected. Therefore, it is a critical public health problem. NAFLD can lead to further complications such as non-alcoholic steatohepatitis (NASH), cirrhosis, liver cancer, liver failure, and even cardiovascular diseases. Obesity is considered the main cause of NAFLD and the rapid increase in obesity due to the increased prevalence of a sedentary lifestyle and high consumption of fast food has led to an enormous rise in the number of patients with NAFLD. Therefore, there is an urgent need to develop new pharmacological interventions that can provide effective and safe treatment for this disease.

Currently, the number of clinically approved pharmacological interventions that can be used in the management of NAFLD, its complications, and predisposing factors such as obesity is highly limited. Moreover, there are concerns regarding the efficacy and safety of the available treatments. In this context, natural products can serve as a vast platform that may provide a good alternative with comparable safety but lower costs. Also, there is an increasing effort in research of natural products which could be of vital importance for protection against and treatment of obesity, NAFLD, and associated cardiovascular complications. The results observed in these studies are promising and show high efficacy and safety for pharmacological use.

The scope of this Research Topic covers these different subjects in four articles—one systematic review, two original articles, and one study protocol. In the first review article, Peng et al. published an interesting systematic review evaluating the effectiveness and safety of Chinese Herbal Medicine (CHM) for type 2 Diabetes mellitus (T2DM) with non-alcoholic fatty liver disease (NAFLD). Several electronic databases were thoroughly searched till December 2021. The authors originally assessed 783 relevant papers found with this search approach. Eighteen studies were finally included in this meta-analysis after duplicates were eliminated and many publications that did not satisfy the required standards were excluded. The 18 studies involved 1,463 patients, 733 in treatment and 730 in control groups. All these studies were randomized control trials (RCTs) carried out in China between 2010 and 2021. The study outcomes were lipid indices, liver functions, insulin, glycemic indices, and body mass index (BMI). These included triglyceride (TG), total cholesterol (TC), low-density lipoprotein cholesterol (LDL-C), high-density lipoprotein cholesterol (HDL-C), alanine transaminase (ALT), aspartate transaminase (AST), homeostatic model assessment of insulin resistance, fasting blood glucose, 2-h postprandial glucose, and the body mass index. The main results of this meta-analysis indicated that the combination of Chinese herbal medicine (CHM) and western medicine (WM) in T2DM patients with NAFLD appears to be more beneficial in improving lipid and glucose metabolism, liver function and insulin resistance, as well as improving overall efficiency and weight loss. The results of this study may provide new treatment options, new ideas, and new directions for the study of T2DM combined with NAFLD.

In the first original article; Wang et al., aimed to investigate how active peptides control the progression of hyperlipidemia. The authors isolated the active peptide AR-9 from *Eupolyphaga sinensis* with the aid of ion exchange chromatography and determined its structure (amino acid sequence) using LC-MS/MS. In order to study AR-9 hypolipidemic action, four groups (model, simvastatin, total *Eupolyphaga*’s active peptides, and AR-9) of rats fed with a high-fat diet were used in addition to a control group that was fed with a normal diet. After 3 weeks of treatment, fresh feces, blood, and liver samples were collected from the different groups for intestinal microbiota, biochemical, histopathological, and metabolomics investigation. It was obvious that the high-fat diet altered the intestinal microbiota as well as the lipid profile in the blood and increased lipid accumulation in the liver. AR-9 succeeded to restore gut beneficial bacteria and reduce fat in obesity-associated rats. Moreover, AR-9 was able to reduce the increased levels of TC, TG, and LDL-C and elevate the decreased level of HDL-C. As for liver tissues, severe microvascular and hepatic steatosis and elevated lipid deposition were recorded in the high-fat diet model group. Treatment with AR-9 reduced lipid deposition and alleviated hepatic steatosis and liver injury. The metabolomics analysis revealed significant changes in the levels of 31 metabolites between the control and model groups. AR-9 was able to increase the level of 11 of the rats. Importing the results to the MetaboAnalyst database indicated that several pathways were disturbed in response to a high-fat diet with arginine biosynthesis as the main pathway. The results from this study shed light on the role and importance of active peptides like AR-9 in the treatment of hyperlipidemia and its associated complications in the liver and gut.

In the second original article; Yin et al. showed that Shuangyu Tiaozhi decoction (SYTZD) alleviates non-alcoholic fatty liver disease by causing decreases in the serum lipids, hepatic enzyme levels, inflammatory cytokines, and homeostatic model assessment for insulin resistance (HOMA-IR). Furthermore, SYTZD treatment affected relative mRNA and protein levels associated with various pathways of NAFLD pathogenesis.

In the study protocol article published by Xu et al.‘s group, the authors evaluated the efficacy and mechanism of the action of the Chinese herbal medicine, Jiedu Tongluo Tiaogan Formula (JTTF), in treating type 2 diabetes mellitus (T2DM) combined with NAFLD. The authors hypothesized that patients will benefit from JTTF, which may provide strong evidence for the clinical use of JTTF in the treatment of T2DM and NAFLD, leading to the possibility of further mechanistic exploration.

